# The Treatment of Abscess in Pott's Disease

**Published:** 1895-11-02

**Authors:** A. H. Tubby

**Affiliations:** Assistant Surgeon and in charge of Orthopædic Department, Westminster Hospital; Surgeon to the National Orthopædic Hospital and the Evelina Hospital for Sick Children.


### MEDICAL PROGRESS AND HOSPITAL CLINICS.

[*The Editor will be glad to receive offers of co-operation and contributions from members of the profession. All letters should be addressed to* The Editor, at the Office, 428, Strand, London, W.C.]

### THE TREATMENT OF ABSCESS IN POTT'S DISEASE.

By A. H. Tubby, M.S.Lond., F.R.C.S.Eng., Assistant Surgeon and in charge of Orthopædic Department, Westminster Hospital; Surgeon to the National Orthopædic Hospital and the Evelina Hospital for Sick Children.

Abscess complicating Pott's disease constitutes a grave source of danger of life. There can be no doubt that such abscesses and their sequelæ are the most frequent cause of death in spinal caries. The form of abscess may be psoas, lumbar, iliac, and pelvic. In gravity they may vary from a small collection of pus coming to the surface as directly as possible from the site of the bone disease, and readily amenable to treatment, to enormous cavities containing pints, and extending beneath muscles and between planes of connective tissue in so devious a manner, as to baffle all attempts at radical treatment. When such abscesses burst they form several discharging sinuses. One or more of these occasionally heal, and a new outlet is formed for the pus at some other spot, perhaps not so favourably situated for drainage and antisepsis as the previous opening. There is no type of case so formidable to the surgeon as this. To give details of such an one:—

Case—M. P., aged 12: On admission there is extensive angular deformity occupying the dorsal region from the seventh to the twelfth dorsal vertebræ. The boy is sallow and cachetic. Pus is discharging from one opening in the right iliac fossa, and from a second on the outer side of the thigh two inches below the trochanter major. Fluctuation extends on the right side from the crest of the ilium over the buttock to the thigh just above the second sinus, and laterally from the anterior to nearly as far as the posterior superior iliac spine; it is also felt over an oval area of about one inch in its long diameter just above the middle of the iliac crest, and again doubtfully one inch external and to the right of the last dorsal vertebra. On pressure over the right iliac fossa curdy pus wells up. An attempt was made to cleanse the discharging sinuses and cavities. The collection in the gluteal region was freely evacuated. Great difficulty was experienced in finding the communication between the smaller collection above the crest of the ilium and that in the gluteal region, but it was subsequently effected. A second incision was made in the space between the last rib and the iliac crest; but the abscess cavity could not be tracked further. Looking at the large extent of the deformity and its general rounded appearance, it was evident that several vertebræ were extensively diseased. It was probable that pus had made its way downwards from the dorsal spine to the crest of the ilium, and had then extended in two directions, externally into the gluteal region, and internally to the iliac fossa. After opening the posterior collections freely, and rubbing and sponging the abscess walls, the sinuses were dressed carefully. It was out of the question to attempt any radical treatment of an abscess extending over such a large area, and with such extensive disease of the vertebræ.

Cases such as the one I have quoted require great skill in dealing with them. Mr. Symonds,[1] speaking in the discussion at the British Medical Association Meeting on the treatment of spinal abscess, quoted the following details: He alluded to a case in which he had opened a large psoas abscess in the thigh, groin, and lumbar region, and obtained primary union. A sinus formed later, and a second abscess was discovered on the other side. The sinus was scraped, the second abscess cleaned out, and the patient was quite well. Lumbar and iliac abscesses constitute a more formidable danger than even psoas abscesses, the possibilities of their rupture into dangerous regions are greater, and their future course is more uncertain. It often happens that they sink into the pelvis, and burst into the rectum or the bladder, or in the perinæum.[2]

Such is the worst side of the picture. Fortunately, there is a more favourable aspect. The methods of treatment open to us are five: (1) The expectant, leaving the abscess to become encysted or absorbed; (2) aspiration; (3) aspiration with the injection of fluids; (4) incision and drainage, with or without washing out the cavity with antiseptics; (5) the method advocated by Treves, Barker, and others; (6) complete removal of the sac by dissection.

Dr. Townsend,[3] of New York, has carefully tabulated the results of treatment of seventy-five cases of spinal abscesses, and I take the liberty of producing his figures in full. The value of the table would have been enhanced if the position of the abscesses had been stated.

Analysis of Seventy-five Cases of Abscesses in Pott's Disease (Townsend).

| | | |
|---|---|---|
| *Expectant Method.* | | |
| No treatment by brace: Abscess disappeared ... ... | 3 | |
| No treatment by brace: Abscess *in statu quo* ... ... | 8 | |
| No treatment by brace: Abscess increasing, child doing well ... ... ... ... ... ... ... ... ... | 8 | |
| No treatment by brace: Abscess increasing, child doing badly ... ... ... ... ... ... ... ... | 2 | |
| | | —21 |
| *Aspirations.* | | |
| Abscesses disappeared after aspiration ... ... ... | 11 | |
| Abscesses opened spontaneously after aspiration failed | 3 | |
| Abscesses incised after aspiration failed ... ... ... | 4 | |
| Abscess *in statu quo* after aspiration failed ... ... | 1 | |
| | | —19 |
| Number of aspirations in each case, from 2 to 6; average 3. | | |
| *Incisions and Scraping of Sac.* | | |
| With use of iodoform emulsion or peroxide of hydrogen:— | | |
| Results—Good ... ... ... ... ... | 11 | |
| Bad ... ... ... ... ... | 3 | |
| *Opened Spontaneously.* | | —14 |
| Results—Good ... ... ... ... ... | 15 | |
| Bad ... ... ... ... ... | 6 | |
| | | —21 |
| | | — |
| | | 75 |
| *Deaths.* | | |
| Tuberculous meningitis ... ... ... ... ... | 2 | |
| Lardaceous disease ... ... ... ... ... | 2 | |
| Suppression of urine ... ... ... ... ... | 1 | |
| | — | |
| | 5 | |

**Absorption of Abscess.—The Expectant Plan.**—Formerly there were but two methods of treatment, to leave the abscess alone, or allow it to burst. That abscesses do disappear gradually no one is prepared to deny. Such may or may not have given rise to symptoms. We may take it that the course of events when pus has formed is as follows: The patient is placed at rest, the spinal column is fixed, the intervertebral pressure is relieved, and pus ceases to be formed. Gradual absorption of the fluid part of the abscess takes place, and there results a cheesy mass, which becomes firmer and tougher, and may partially or entirely calcify, and be surrounded by a firm fibrous capsule. In the non-calcareous parts and in the outlying parts of the capsule numerous tubercle bacilli are found. Their presence always constitutes a distinct menace to health and life, and may at any time light up fresh local, or cause general tuberculosis.

The indications for the expectant treatment are:—

1. When the abscess is apparently single, and not tracking in two or more directions. 2. When the recumbent position is followed immediately by cessation from pain, and improvement of the general health. 3. The expectant plan should be persevered with, when, after a short trial, the abscess ceases to enlarge. 4. If it is evident that pus is near the skin and pointing, it is better to open antiseptically and so avoid the risks of spontaneous opening. 5. A large collection of pus is no hindrance to the trial of this method, provided that the appetite is good and the temperature is normal; in fact in those abscesses which were formerly designated as "cold." At the present moment I am watching the course of a large lumbar and iliac abscess.

Case: A girl, aged eight years, was admitted to the National Orthopædic Hospital on December 19th, 1893, with a posterior projection of the spine extending from the tenth dorsal to the first lumbar vertebræ. A large abscess was felt in the right iliac fossa. It reached internally as far as the umbilicus, and fluctuation was manifest on the outer side of the femoral artery in Scarpa's triangle. She was placed in bed, extension applied, and given cod-liver oil. On February 2nd, 1894, the abscess was found to have diminished sensibly, and did not now extend beyond the mid-point of a line drawn from the anterior superior iliac spine to the umbilicus, and there was corresponding decrease in other dimensions. On April 30th all fluctuation had disappeared from below Poupart's ligament, and the abscess was limited to the iliac fossa. The child is now fat and healthy, and has put on fifteen pounds in weight.

But unfortunately good results on the expectant plan are the exception and not the rule. Apparent diminution in the abscess may take place from the pus sinking into the pelvis. If absorption takes place the result for the immediate present may be regarded as satisfactory, but there is always a possibility of a recrudescence of the tubercular process. In any case, time is gained, and when the abscess lights up again, as so many do, opportunity is afforded for other and more radical modes of treatment. Personally I should be inclined, if the indications mentioned above are present, to give the patient the chance of absorption on the expectant plan. One's hand is still free to adopt other measures later.

